# Methyl Orange-Doped Polypyrrole Promoting Growth of ZIF-8 on Cellulose Fiber with Tunable Tribopolarity for Triboelectric Nanogenerator

**DOI:** 10.3390/polym14020332

**Published:** 2022-01-14

**Authors:** Qiang Li, Xianhui An, Xueren Qian

**Affiliations:** Key Laboratory of Bio-Based Material Science & Technology, Northeast Forestry University, Ministry of Education, Harbin 150040, China; 2020115563@nefu.edu.cn (Q.L.); anxianh509@163.com (X.A.)

**Keywords:** cellulose fiber, polypyrrole, methyl orange, ZIF-8, mediating layer, triboelectric nanogenerator

## Abstract

Cellulose fiber (CelF) is a biodegradable and renewable material with excellent performance but negligible triboelectric polarizability. Methods to enhance and rationally tune the triboelectric properties of CelF are needed to further its application for energy harvesting. In this work, methyl-orange-doped polypyrrole (MO-PPy) was in situ coated on CelF as a mediating layer to promote the growth of metal–organic framework ZIF-8 and to construct a cellulose-based triboelectric nanogenerator (TENG). The results showed that a small amount of MO-PPy generated in situ significantly promoted the growth of ZIF-8 on CelF, and the ZIF-8 deposition ratio was able to increase from 7.8% (ZIF-8/CelF) to 31.8% (ZIF-8/MO-PPy@CelF). ZIF-8/MO-PPy@CelF remained electrically conductive and became triboelectrically positive, and the triboelectricity’s positivity was improved with the increase in the ZIF-8 deposition ratio. The cellulose-based TENG constructed with ZIF-8/MO-PPy@CelF (31.8% ZIF-8 deposition ratio) and polytetrafluoroethylene (PTFE) could generate a transfer charge of 47.4 nC, open-circuit voltage of 129 V and short-circuit current of 6.8 μA—about 4 times higher than those of ZIF-8/CelF (7.8% ZIF-8 deposition ratio)—and had excellent cycling stability (open-circuit voltage remained almost constant after 10,000 cycles). MO-PPy not only greatly facilitated the growth of ZIF-8 on CelF, but also acted as an electrode active phase for TENG. The novel TENG based on ZIF-8/MO-PPy@CelF composite has cheerful prospects in many applications, such as self-powered supercapacitors, sensors and monitors, smart pianos, ping-pong tables, floor mats, etc.

## 1. Introduction

In the past few decades, due to the significant increase in energy consumption, people have paid more attention to renewable energy. Finding renewable energy to reduce carbon emissions, ensure long-term energy supply and reduce dependence on fossil fuels is the inevitable requirement of sustainable economic development. Various methods have been proven to be able to convert renewable energy [1,2,3,4,5,6,7,8,9]. Among energy harvesting technologies, the triboelectric nanogenerator (TENG), based on the combination of triboelectrification and electrostatic induction, was fabricated for the efficient conversion of various forms of green and renewable energy into electrical energy [10,11,12,13,14]. TENG operates based on the contact of two different friction materials with opposing tendencies of electron affinity. More precisely, triboelectrification supplies opposite polarization charges on the surface of each contact material, and mechanical energy is then converted into electrical energy driven by electrostatic induction [15]. In addition to this facile operation mode, TENG has many advantages such as light weight, high cost–benefit, facile manufacturing and high output voltage at low frequencies [16,17]. Thus, TENG is a promising technology in the field of energy harvesting.

At present, most of the raw materials of TENG are non-renewable synthetic polymers, which meet the performance requirements of TENG [18,19]. However, these unsustainable materials cannot degrade naturally and have an adverse impact on the environment in long-term application, which also brings some problems to TENG as a more environmentally friendly and feasible energy acquisition device. Considering the environment and health and safety issues, it is very important to use sustainable and environmentally friendly materials to manufacture TENG. Cellulose-based materials with good biocompatibility and biodegradability can be in close contact with biological systems without any adverse or toxic reactions. Their application in the field of TENG can not only realize the function of collecting environmental energy, but also ensure the environmental protection of TENG in material application. In recent years, cellulose-based TENG (C-TENG) has attracted more and more attention [20,21,22,23,24,25,26,27,28,29,30,31]. Looking back to the overall development of C-TENG, we find that the main strategy to improve the output performance of C-TENG is realized by modifying cellulose-based materials in addition to optimizing TENG’s structure [32,33,34,35]. It is critical for improving the output property to adjust the triboelectric polarity of cellulose-based materials by physical and chemical modification. On the other hand, optimizing the morphology of cellulose-based materials to increase the effective contact area is also a key to improving the output performance of C-TENG. However, so far there are few cases of simultaneous optimization of either triboelectric polarity or morphology of cellulose-based materials.

Metal–organic frameworks (MOFs) are porous materials formed by the combination of organic ligands and metal ions. Due to the flexibility of MOFs in composition, size and function, they have been determined as very promising triboelectric materials [36,37]. In particular, ZIF-8 has been proven to exhibit positive triboelectric behavior [36]. In addition to its positive triboelectric behavior, its unique size adjustability and particle geometry—thus adjusting the nano-roughness of ZIF-8-functionalized triboelectric surfaces—make it a very promising candidate for the development of C-TENG. However, as the functional groups of untreated cellulose fiber (CelF) are mainly alcohol hydroxyl groups, they have limited ability to adsorb and chelate metal ions, which makes them difficult to grow and efficiently anchor ZIF-8 on CelF. Some studies were conducted to promote the loading of MOFs on CelF [38,39,40,41,42,43,44,45]. Polypyrrole (PPy), as a common conductive polymer, has the advantages of good stability, high conductivity, easy synthesis, environmental friendliness, particularly low cost and strong ability to adsorb metal ions [46]. Therefore, it can be used as a mediating layer to facilitate the growth and anchoring of ZIF-8 on CelF. More importantly, the composite material deposited both PPy and ZIF-8 remains electrically conductive and can therefore be used not only as a positive triboelectric layer but also as an electrode without needing to integrate an additional electrode.

**Scheme 1:**
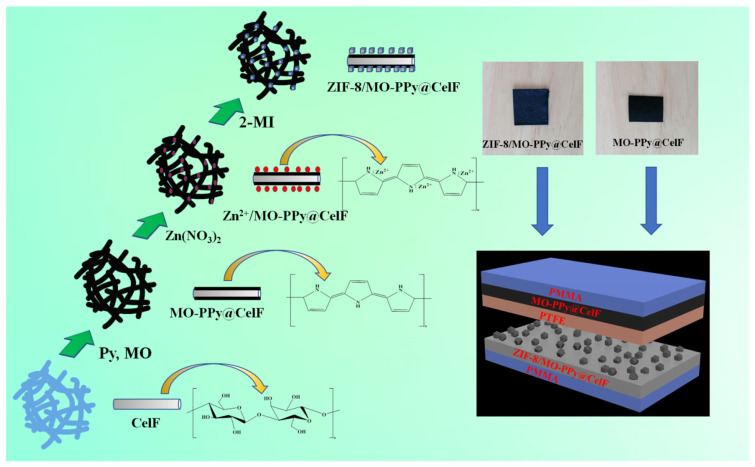
Schematic illustration of preparation and application of ZIF-8/MO-PPy@CelF composite.

In this work, we propose a simple method for the efficient growth of ZIF-8 on the surface of CelF using methyl-orange (MO)-doped PPy (MO-PPy) layer as an intermediary between CelF and ZIF-8, thereby achieving efficient loading and interfacial binding of ZIF-8 on CelF. The principle and process for the preparation of ZIF-8/MO-PPy@CelF composite is displayed in Scheme 1. ZIF-8 particles were grown and immobilized on the surface of MO-PPy/CelF through the strong coordination interaction between amino (NH) groups in MO-PPy and Zn^2+^. The NH groups of PPy itself have a chelating effect on Zn^2+^, while the doped MO with azo groups also has an adsorption effect on Zn^2+^, thus making ZIF-8 grow more firmly on the CelF surface. Finally, the synthesized composite material was made into sheet material and used for the subsequent fabrication of C-TENG. The promoting effect of MO-PPy on ZIF-8 growth was demonstrated, the resulted ZIF-8/MO-PPy@CelF composite was characterized by SEM, XRD, FTIR and XPS, and the effect of ZIF-8 deposition ratio on the open-circuit voltage, transfer charge and short-circuit current of C-TENG was investigated. The resulting optimized functional C-TENG showed more than 4 times higher triboelectric electrical output than unmodified C-TENG.

## 2. Experimental Section

### 2.1. Materials and Reagents

CelF (bleached kraft softwood pulp) was obtained from Mudanjiang Hengfeng Paper Co., Ltd. (Mudanjiang, China) and beaten to 35 °SR before use. F5 LED lamp beads were produced by Shenzhen Keweijingxin Technology Co., Ltd. (Shenzhen, China). Pyrrole (Py, analytical purity) was produced by Sinopharm Chemical Reagent Co., Ltd. (Shanghai). Ferric chloride hexahydrate (FeCl_3_ · 6 H_2_O) was produced by McLean Reagent Co., Ltd. (Shanghai, China). MO was obtained from Shandong Xiya Chemical Industry Co., Ltd. (Linyi, China). Zinc nitrate hexahydrate (Zn(NO_3_)_2_ · 6 H_2_O, 99.0%) was produced by Shanghai Macklin Biochemical Co., Ltd. (Shanghai, China). 2-Methylimidazole (2-MI, 98.0%) was produced by Aladdin Biochemical Technology Co., Ltd. (Shanghai, China). With the exception of Py monomer, which was distilled under reduced pressure before use, all chemicals were directly used without further purification.

### 2.2. Preparation of CelF-Based Composites

#### 2.2.1. Preparation of MO-PPy@CelF Composite

MO-PPy@CelF composite was prepared by in situ polymerization process. First, 2 g of oven-dry CelF was placed in 150 mL distilled water, and 0.1 g of MO was added. Then, 1 mL of Py was added to the above mixture. Next, 4.1 g of FeCl_3_ · 6H_2_O, dissolved in 50 mL of distilled water, was slowly added to the mixture and stirred continuously at 0–5 °C for 2 h. Finally, MO-PPy@CelF composite was obtained by washing with distilled water. For comparison, a PPy@CelF composite without the addition of MO was also prepared.

#### 2.2.2. Preparation of ZIF-8@CelF Composite

ZIF-8@CelF composite was synthesized by simple in situ growth method. First, 2 g of CelF (oven-dry) was added to 150 mL of mixed solvent (the volume ratio of water and methanol is 1:1) containing a certain amount of Zn(NO_3_)_2_ · 6 H_2_O for 3 h. Then, 2-MI (the molar ratio of 2-MI to zinc nitrate is 4:1), dissolved in 50 mL of mixed solvent, was added and stirred magnetically. Finally, ZIF-8@CelF composite was obtained by washing and drying at 60 °C.

#### 2.2.3. Preparation of ZIF-8/MO-PPy@CelF Composite

ZIF-8/MO-PPy@CelF composite was prepared by double in situ method. First, 2 g of CelF (oven-dry) and 0.1 g of MO were added to 150 mL distilled water. Then, 1 mL of Py was added to the above mixture. 4.1 g of FeCl_3_ · 6 H_2_O, dissolved in 50 mL of distilled water, was slowly added to the mixture and stirred continuously at 0–5 °C for 2 h. MO-PPy@CelF composite was obtained by washing with distilled water. Next, 2 g of MO-PPy@CelF (oven-dry) was added to 150 mL of mixed solvent (the volume ratio of water to methanol is 1:1) containing a certain amount of Zn(NO_3_)_2_ · 6 H_2_O and stirred for 3 h. Then, 2-MI (the molar ratio of 2-MI to zinc nitrate is 4:1), dissolved in 50 mL of mixed solvent, was added and stirred magnetically. ZIF-8/MO-PPy@CelF was obtained by washing with distilled water. The uniformly dispersed ZIF-8/MO-PPy@CelF suspension was poured into a sand core funnel fitted with filter paper. After vacuum filtration, the wet ZIF-8/MO-PPy@CelF paper sheet was removed and dried at 105 °C for 16 min (8 min each side). The thickness of the ZIF-8/MO-PPy@CelF composite paper was measured using an IMT-HD02 thickness tester produced by Dongguan Gaoxin Testing Equipment Co., Ltd. (Dongguan, China).

### 2.3. Calculation of ZIF-8 Deposition Ratio

The as-synthesized simple was dried at 105 °C for 3 h. After cooling for 30 min in a dryer, the mass was measured. The deposition ratio (*D*, %) of ZIF-8 was calculated using the following formula:

*D* = (*M*_2_ − *M*_1_)/*M*_0_ × 100% (1)

where *M*_0_ is the original mass of CelF, g; *M*_1_ is the mass of the composite coated MO-PPy, g; *M*_2_ is the mass of the composite deposited ZIF-8, g.

### 2.4. Fabrication of C-TENG

Polytetrafluoroethylene (PTFE) film (4 × 4 cm^2^) was used as the negative friction layer. The ZIF-8/MO-PPy@CelF (4 × 4 cm^2^) was used as the positive friction layer and electrode material. MO-PPy@CelF was used as a bottom electrode, attached on bottom of a PTFE film. Polymethyl methacrylate (PMMA) boards were cut and used as support materials for the TENG. Top and bottom parts were attached to two PMMA boards. The positive and negative copper wires were connected to the friction material and the electrode, respectively. Positive and negative copper wires were then connected to a Keithley 6514 electrometer produced by Keithley Instruments, Inc. (Cleveland, OH, USA) to test the output performance of the TENG. A JZK-10 modal exciter produced by Jiangsu Lianneng Electronic Technology Co., Ltd. (Yangzhou, China) was used as an external force source. In this work, the force exerted by the contact separation was 50 N, and the frequency during the experiment was 2 Hz.

### 2.5. Characterization

The morphology of samples was analyzed using a Zeiss sigma 300 scanning electron microscope (Carl Zeiss AG, Oberkochen, Germany) with intelligent EDX spectrometer at an accelerating voltage of 0.02–30 kV. Before observation, the sample surface was coated with gold under vacuum. The wavelength of the nickel-filtered Cu-Kα radiation source was 1.5418 Å, the voltage was 40 kV and the current was 40 mA. Thermo Scientific Nicolet iS5 FTIR spectrometer (Thermo Fisher Scientific, Waltham, MA, USA) was used to record the FTIR spectra of samples in the frequency range of 4000–400 cm^−1^. The element composition and valence information of samples was measured using ESCALAB 250xi X-ray photoelectron spectrometer (Thermo Fisher Scientific, MA, USA) with Al-Kα radiation source (*hv* = 1486.6 eV). RIGAKU Ultima IV X-ray diffractometer (Beijing Guanyuan Technology Co., Ltd., Beijing, China), with a scanning range of 5–90° and scanning speed of 5°/min, was used to analyze the crystalline nature of the samples. The Kelvin probe force microscopy (KPFM) measurement was taken on an AFM system using Pt/Ir-coated SCM-PIT probe (Bruker Corporation, Billerica, MA, USA). RTS-8 four probes tester (Guangzhou Four Probe Technology Co., Ltd., Guangzhou, China) was used to determine the conductivity of the samples.

## 3. Results and Discussion

### 3.1. Promoting Effect of MO-PPy on the Growth of ZIF-8

**Figure 1:**
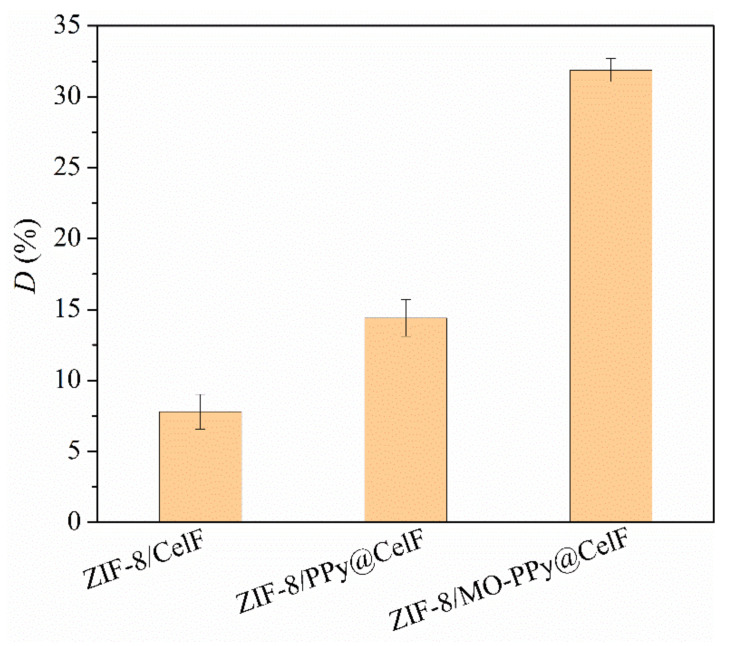
Comparison of ZIF-8 growth on three CelF substrates. Process conditions: 25 °C, Py 1 mL, Zn(NO_3_)_2_ 12 mmol, 6 h.

As shown in Figure 1, the ZIF-8 deposition ratio on CelF was effectively increased up to 31.8% when MO-PPy was used as the mediating layer. In contrast, without PPy, the deposition ratio of ZIF-8 on CelF was only 7.8%. When using PPy without MO doping, the deposition ratio of ZIF-8 was 14.4%, indicating that the MO-PPy in situ coated as a mediating layer can significantly promote the growth of ZIF-8 on CelF. The promoting effect of MO-PPy on the growth of ZIF-8 on CelF was mainly attributed to the adsorption of metal ions by NH groups of PPy [47] and azo groups of MO molecules [48].

**Figure 2:**
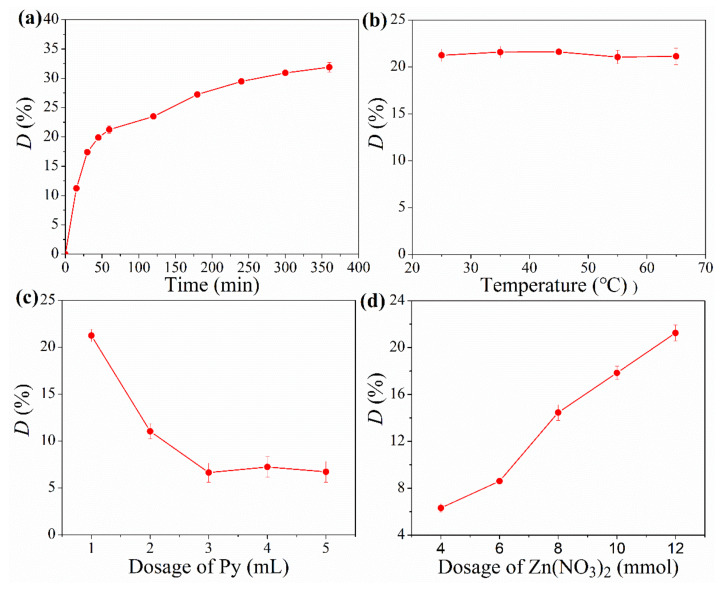
Effect of time (**a**), temperature (**b**), dosages of Py (**c**) and Zn(NO_3_)_2_ (**d**) on ZIF-8 deposition. Process conditions: (**a**) 25 °C, Py 1 mL, Zn(NO_3_)_2_ 12 mmol; (**b**) Py 1 mL, Zn(NO_3_)_2_ 12 mmol, 1 h; (**c**) 25 °C, Zn(NO_3_)_2_ 12 mmol, 1 h; (**d**) 25 °C, Py 1 mL, 1 h.

The effects of process variables such as temperature, time, Py and zinc nitrate dosages on the deposition ratio of ZIF-8 on CelF were also investigated. As shown in Figure 2a, time had a noticeable effect on the deposition ratio of ZIF-8. The deposition ratio of ZIF-8 increased rapidly in the first hour, after which it increased slowly. The deposition ratio of ZIF-8 reached 31.8% at 6 h. At the initial stage, the amount of ZIF-8 deposited was low for a short period of time as the added metal ions had not yet fully reacted with the ligand, which was attributed to the low amount of nucleation of ZIF-8 at this time. The deposition ratio of ZIF-8 increased significantly with the prolongation of time, which was attributed to the full reaction of the metal ions with the ligand. Temperature had almost no influence on the deposition of ZIF-8 on CelF (Figure 2b), indicating that ZIF-8 in situ growth has an extremely low activation energy. As shown in Figure 2c, ZIF-8 deposition ratio decreased with Py dosage; its value was the highest when Py dosage was 1 mL, indicating that a small amount of MO-PPy as a mediating layer can promote the growth of ZIF-8 on CelF, and the thick MO-PPy coating is not conducive to the deposition of ZIF-8. The deposition ratio of ZIF-8 increased with increasing zinc nitrate dosage, reaching a deposition ratio of 21.2% when zinc nitrate dosage was 12 mmol (Figure 2d).

### 3.2. Morphology and Structure of Composites

#### 3.2.1. SEM Observation

**Figure 3:**
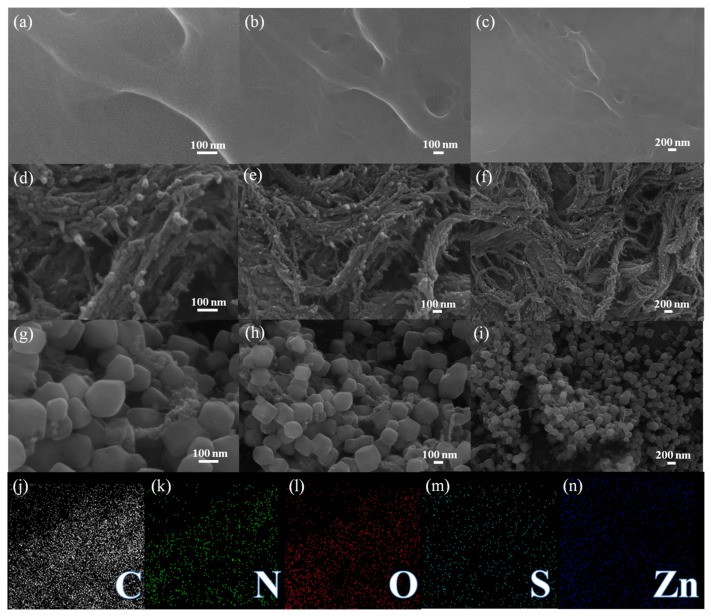
SEM images of CelF (**a**–**c**), MO-PPy@CelF (**d**–**f**), ZIF-8/MO-PPy@CelF (**g**–**i**); EDS images of ZIF-8/MO-PPy@CelF (**j**–**n**).

Figure 3 shows SEM images of CelF, MO-PPy@CelF and ZIF-8/MO-PPy@CelF with different magnifications. The untreated CelF has a relatively smooth and flat surface (Figure 3a–c). In fact, the smooth surface of CelF is not conducive to the deposition of MOF. The fibrous MO-PPy attached to the surface of CelF could be observed in Figure 3d–f, resulting in a rough and uneven cellulose surface with many micropores, which greatly facilitated the efficient growth of ZIF-8. As shown in Figure 3g–i, ZIF-8 nanoparticles with sizes of about 150–200 nm were uniformly grown on the surface of MO-PPy@CelF. With the incorporation of ZIF-8, CelF obtained a rougher surface, which played a key role in the TENG output. As shown in Figure 3j–n, the element maps of ZIF-8/MO-PPy@CelF showed the uniform distribution of C, N, O, S and Zn elements on the surface of the composite, which further indicated the successful synthesis of ZIF-8 on the surface of MO-PPy@CelF. Meanwhile, the topographic SEM images of ZIF-8/MO-PPy@CelF were also analyzed. At positions 1 (Figure S1a,b) and 2 (Figure S1c,d), it can be observed that ZIF-8 nanoparticles grew not only on the surface of MO-PPy but also within the interwoven network of pores formed by MO-PPy. At position 3 (Figure S1e), the interfacial adhesion between cellulose and ZIF-8 can be observed.

#### 3.2.2. XRD Analysis

**Figure 4:**
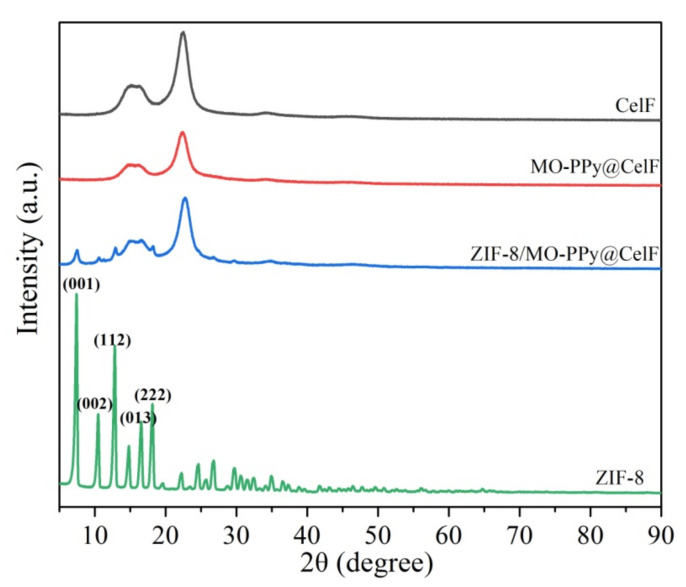
XRD curves of CelF, MO-PPy@CelF, ZIF-8 and ZIF-8/MO-PPy@CelF.

The XRD patterns of CelF, MO-PPy@CelF, ZIF-8 and ZIF-8/MO-PPy@CelF are shown in Figure 4. Both CelF and MO-PPy@CelF displayed the characteristic peaks of cellulose I with a sharp high-intensity peak (200) centered at 2θ = 22.5°; two peaks at 14.9° and 16.4° can be attributed to CelF [49]. The comparison of XRD patterns of CelF and MO-PPy@CelF shows that the coverage of amorphous MO-PPy on the surface of CelF does not change the crystal structure of CelF. Compared to CelF and MO-PPy@CelF, the peaks of ZIF-8/MO-PPy@CelF at 2θ = 7.3°, 10.4°, 12.7°, 14.7°, 16.4° and 18.0° associated to (011), (022), (112), (022), (013) and (222) of ZIF-8 crystal planes [50]. The above results confirmed that ZIF-8 nanocrystals were generated from 2-MI and Zn^2+^, resulting in the formation of ZIF-8/MO-PPy@CelF composite material.

#### 3.2.3. FTIR Analysis

**Figure 5:**
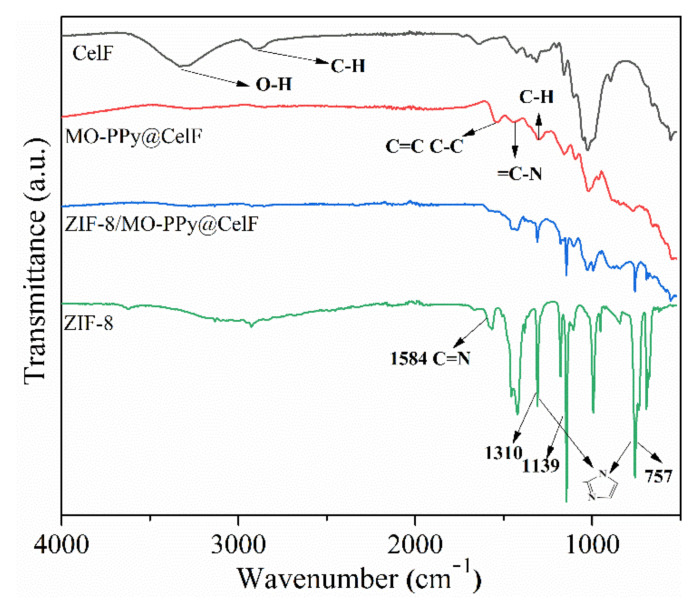
FTIR spectra of CelF, MO-PPy@CelF, ZIF-8 and ZIF-8/MO-PPy@CelF.

The FTIR spectra of CelF, MO-PPy@CelF, ZIF-8 and ZIF-8/MO-PPy@CelF were recorded; the results are displayed in Figure 5. The spectrum of CelF had obvious characteristic peaks of cellulose. The wide peak at about 3300 cm^−1^ was attributed to the O–H stretching vibration, and the peak at about 2990 cm^−1^ was ascribed to the C-H stretching vibration. After the deposition of MO-PPy, the O–H stretching vibration peak at 3300 cm^−1^ and the C–H stretching vibration at 2990 cm^−1^ were significantly weakened, which indicates that the cellulose was completely covered. The absorption peak at about 1630 cm^−1^ in the spectrum of MO-PPy@CelF was assigned to the N–H stretching vibration of PPy, demonstrating the successful decoration of PPy on pure CelF. For ZIF-8, the peak at 1584 cm^−1^ corresponded to the C=N stretching of the imidazole ring [50,51]; the convoluted peaks at 1500–600 cm^−1^ were attributed to the entire imidazole ring stretching or bending effects [52]. It can be found that the FTIR spectrum of ZIF-8/MO-PPy@CelF contained the characteristic peaks of CelF and ZIF-8. The characteristic peaks at 1139, 1310 and 757 cm^−1^ in the ZIF-8 appeared in ZIF-8/MO-PPy@CelF, indicating the successful growth of ZIF-8 nanoparticles on MO-PPy@CelF.

#### 3.2.4. XPS Analysis

**Figure 6:**
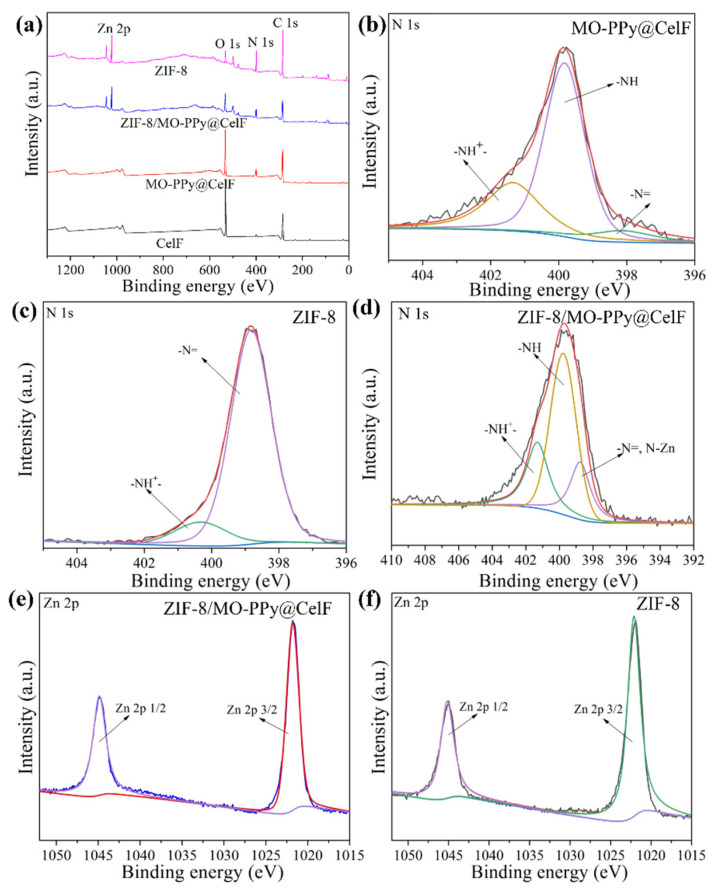
(**a**) XPS wide scan spectra of CelF, MO-PPy@CelF, ZIF-8 and ZIF-8/MO-PPy@CelF; (**b**–**d**) N 1s XPS spectra of MO-PPy@CelF, ZIF-8 and ZIF-8/MO-PPy@CelF; (**e**,**f**) Zn 2p spectra of ZIF-8/MO-PPy@CelF and ZIF-8.

The XPS spectra of CelF, MO-PPy@CelF, ZIF-8 and ZIF-8/MO-PPy@CelF further provided rich information about the chemical state of the surface elements of the composites. As shown in Figure 6a, C and O peaks appeared in all samples, which were attributed to the rich hydroxyl groups in the carbon skeleton of organic compounds and cellulose matrix. The N element peak represents MO-PPy and ZIF-8, and the Zn element peak also represents ZIF-8. The above characteristic peaks appeared in ZIF-8/MO-PPy@CelF, which indicated that both ZIF-8 and MO-PPy were deposited on the surface of CelF, and ZIF-8/MO-PPy@CelF composite was successfully prepared. Figure 6b shows N 1s narrow scan XPS spectrum of MO-PPy@CelF, and the N 1s could be deconvoluted into three peaks of 399.4, 401 and 402.3 eV, corresponding to –NH–, –NH^+^– and –N= bonds, respectively [53]. In the XPS spectrum of ZIF-8, the N 1s can be fitted into two peaks at 401.1 eV and 398.4 eV (Figure 6c), which corresponds to pyrrolic N and pyridinic N, respectively [54]. In the XPS spectrum of ZIF-8/MO-PPy@CelF composite, the N1s was deconvoluted into three peaks (Figure 6d). Two peaks at 398.2 eV and 398.8 eV were associated with the –NH– and –NH^+^– groups of pyrrole unit [55]. The peak at 398.6 eV was attributed to C=N defects of MO-PPy and the coordination of N–Zn [56]. Figure 6e and f show the Zn 2p XPS spectra of ZIF-8/MO-PPy@CelF and ZIF-8, whereas the Zn 2p spectrum can be deconvoluted into two peaks, i.e., 2p1/2 and 2p 3/2, located at 1044.8 eV and 1021.8 eV, indicating Zn^2+^ existed in the ZIF-8/MO-PPy@CelF composite. In conclusion, the XPS results clearly show that the ZIF-8/MO-PPy@CelF composite was successfully prepared.

### 3.3. TENG for Energy Harvesting

#### 3.3.1. Working Principle

**Figure 7:**
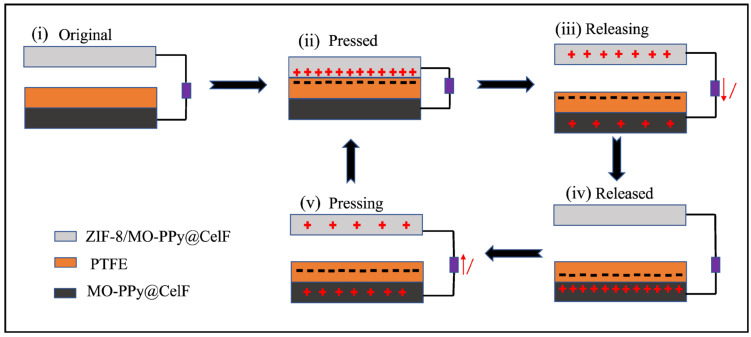
Schematic illustration of the TENG working principle.

**Figure 8:**
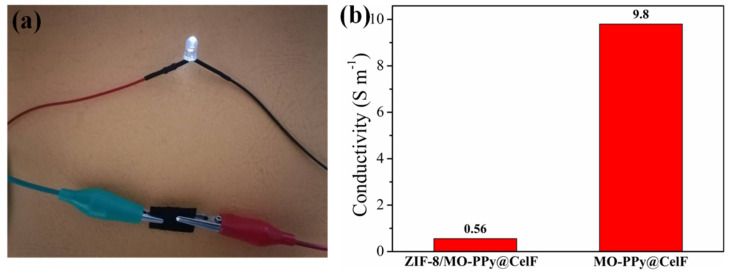
(**a**) Lighting of an LED using the ZIF-8/MO-PPy@CelF with 31.8% ZIF-8 deposition ratio (0.85 mm thick); (**b**) Conductivity values of ZIF-8/MO-PPy@CelF and MO-PPy@CelF.

In a contact-separation operating mode, the working principle of the C-TENG is based on the coupling effect of contact electrification and electrostatic induction [57], as shown in Figure 7. In this work, ZIF-8/MO-PPy@CelF was used as a positive friction layer and electrode material, mainly based on the fact that ZIF-8/MO-PPy@CelF exhibits electrical conductivity (Figure 8). Meanwhile, MO-PPy@CelF with a conductivity of 9.8 S·m^−1^ was used as a bottom electrode, attached on the bottom of the PTFE film. In the initial state, no charges generate on the surface of ZIF-8/MO-PPy@CelF composite or PTFE (Figure 7i). Nevertheless, when the two friction layers are in full contact, static electrostatic charges are generated on the two friction materials driven by external forces (Figure 7ii). Owing *to* the high triboelectric positivity of the ZIF-8/MO-PPy@CelF and the strong triboelectric negativity of the PTFE, these two triboelectric materials tend to lose and gain electrons during contact, respectively. Consequently, the negative charges are generated on the PTFE and the positive charges are inducted on the top ZIF-8/MO-PPy@CelF due to the contact electrification effect. In this state, no electrons flow through the external circuit, because the induced charges of opposite polarity are in equilibrium. Whereas after releasing the external force, the top ZIF-8/MO-PPy@CelF starts to separate from the PTFE, thus resulting to an electrostatic field between the top ZIF-8/MO-PPy@CelF and bottom MO-PPy@CelF (Figure 7iii). The electrostatic field drives the flow of electrons from the bottom MO-PPy@CelF to the top ZIF-8/MO-PPy@CelF, thereby generating a current with an opposite direction. When the triboelectric layer is completely released, the electric signal disappears due to the equilibrium state of the triboelectric charges (Figure 7iv). When the TENG is pressed again, the reduction in the distance between the friction layers leads to an opposite electric potential, thus generating a current flowing from the bottom of the MO-PPy@CelF to the top of the ZIF-8/MO-PPy@CelF (Figure 7v). Modification of CelF surface with ZIF-8 facilitates the accumulation of positive charges, thus increasing charge transfer and improving the output performance of TENG [57].

#### 3.3.2. Output Performance

**Figure 9:**
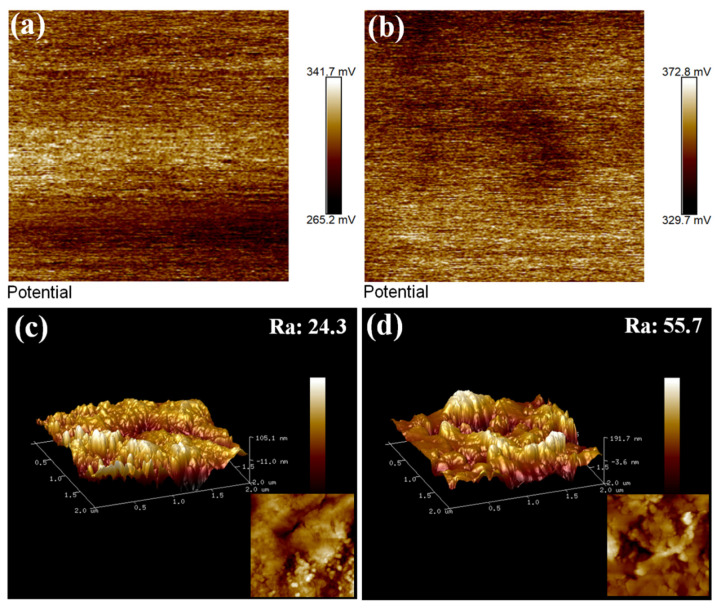
Surface potentials of ZIF-8/MO-PPy@CelF with 17.4% (**a**) and 31.8% (**b**) ZIF-8 deposition ratios; Surface roughness values of ZIF-8/MO-PPy@CelF with 17.4% (**c**) and 31.8% (**d**) ZIF-8 deposition ratios.

The surface potential of friction materials plays a critical role in the output performance of the TENG [36]. Here, the surface potential of ZIF-8/MO-PPy@CelF was studied using KPFM, which proved the positive surface potential of ZIF-8/MO-PPy@CelF (Figure 9a,b). Specifically, a positive contact potential difference, *V*_CPD_, means that electrons can tunnel easily from the surface, i.e., the surface can be positively charged easily when in contact with the other material [36]. Therefore, ZIF-8 can be used as a positive triboelectric material in the TENG. Based on the good performance of ZIF-8, PTFE and ZIF-8/MO-PPy@CelF were selected as the negative and positive friction layers for the fabrication of TENG. The positively charged nature of ZIF-8 may also be related to its internal functional groups. The ZIF-8 structure contains imidazole ligands with –NH functional groups, which tend to contribute electrons, thus making the material containing the –NH functional groups positively charged [58]. Meanwhile, the surface roughness of the friction material also plays an important role in the output performance of TENG [59]. The increase in surface roughness improves the TENG output as it brings more area into contact during the working of the TENG. Figure 9c,d shows the surface roughness values of two ZIF-8/MO-PPy@CelF samples with different ZIF-8 deposition ratios. The ZIF-8/MO-PPy@CelF with 31.8% ZIF-8 deposition ratio is rougher than that with 17.4% ZIF-8 deposition ratio. Therefore, the increase in ZIF-8 deposition ratio is beneficial to the improvement of TENG performance.

**Figure 10:**
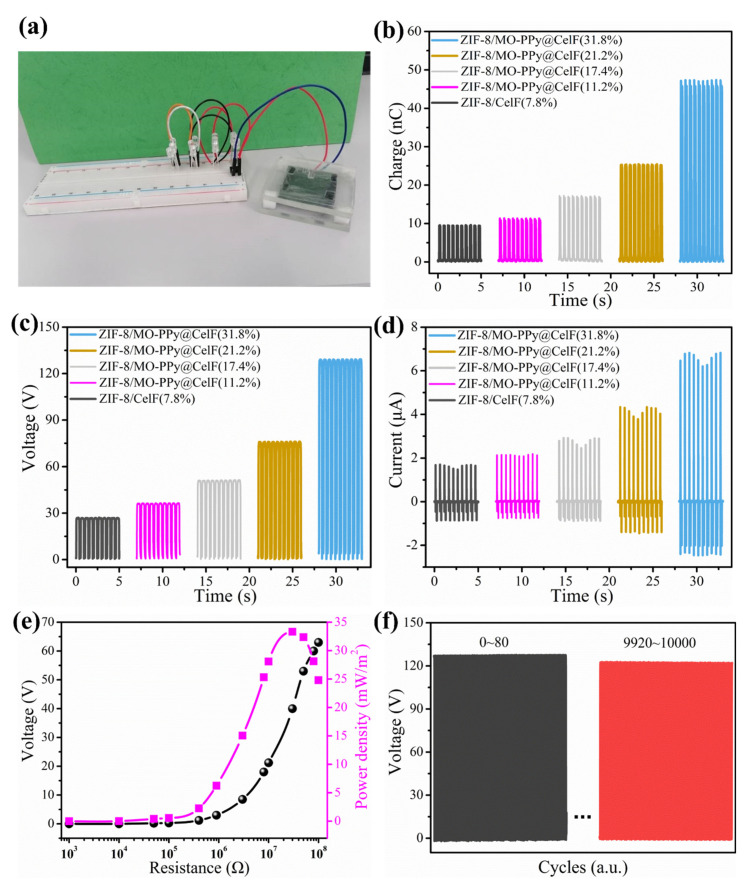
(**a**) Photograph of C-TENG, (**b**) transfer charge, (**c**) open-circuit voltage and (**d**) short-circuit current of C-TENG, (**e**) power output of C-TENG on external load resistances, (**f**) stability test of the C-TENG.

In this work, both the surface potential and roughness of CelF surface can be improved by increasing the deposition ratio of ZIF-8 on CelF surface. Whereas the limited ability of pure CelF to adsorb and chelate metal ions leads to less deposition of ZIF-8 on CelF surface. The MO-PPy generated in situ was used as a mediating layer to increase the deposition ratio of ZIF-8 on CelF surface, thereby improving the performance of C-TENG. Therefore, we focused on the effect of ZIF-8 deposition on the output performance of TENG such as transfer charge, short-circuit current and open-circuit voltage. Figure 10a show an image of the C-TENG. As shown in Figure 10b–d, the transfer charge, open-circuit voltage and short-circuit current of the modified C-TENG increased with an increasing deposition ratio of ZIF-8. When the deposition ratio of ZIF-8 was 31.8%, the transfer charge was the highest (47.4 nC) (Figure 10b). Charge transfer of the triboelectric materials played an important role in the output performance of TENG [60]. The open-circuit voltage increased from 27 V to 129 V (Figure 10c) and the short-circuit current increased from 1.7 μA to 6.8 μA (Figure 10d), approximately four times higher than those of the unmodified C-TENG, as the deposition ratio of ZIF-8 increased from 7.8% to 31.8%. Meanwhile, we calculated the current densities as shown in Figure S2. The good output performance of TENG is mainly attributed to more ZIF-8 deposition on the CelF surface. The more ZIF-8 deposited on the CelF surface, the positive polarity was stronger, and the more surface charge was generated on surface of materials.

As seen in Figure 10e, the voltage increased with increasing external resistance and the output power density increased and then decreased with increasing resistance, reaching a maximum of 33.3 mW·m^−2^ at a resistance of 30 MΩ. Moreover, as shown in Video S1, the C-TENG was connected to several LED lights in series, and 12 LEDs were successfully lit up with the slap of hands. In addition to excellent performance, the operating stability of the TENG is an important indicator in order to ensure the long-term collection of environmental mechanical energy [61]. As shown in Figure 10f, the open-circuit voltage of the C-TENG remained almost constant after 10,000 cycles at a frequency of 2 Hz and an external force of 50 N, indicating the C-TENG had excellent cycling stability. At the same time, due to the hydrophilic nature of cellulose, we found that the output data of TENG decreased at high relative humidity (Figure S3). This work has made some efforts towards the preparation of positive cellulose-based friction materials. The output performance comparison of C-TENG is shown in Table S1 [27,62,63,64,65,66,67]. This work successfully deposited ZIF-8 onto CelF using MO-PPy as the mediating layer and the output performance of the assembled C-TENG can be tuned by the deposition ratio of ZIF-8 on CelF. The preparation of ZIF-8/MO-PPy@CelF in this work is simple and green, and the C-TENG can reach an open-circuit voltage of 129 V and a power density of 33.3 mW·m^−2^, showing good output performance.

As discussed earlier, the ZIF-8/MO-PPy@CelF-based TENG developed by this work was proven to be the excellent power source for lighting LEDs. Cellulose-based TENGs have been reported to provide power for electronics [68,69,70,71,72]. In this work, TENG based on ZIF-8/MO-PPy@CelF can potentially serve as a power source for supercapacitors to store energy. Flexible sensors based on ZIF-8/MO-PPy@CelF-based TENG are worth developing and are expected to enable tactile sensing and monitoring of various physiological signals such as pulses in the human body. TENG based on ZIF-8/MO-PPy@CelF can also be used in emerging smart necessities such as paper-based energy-harvesting pianos, ping-pong tables, floor mats, etc.

## 4. Conclusions

Using MO-PPy as a bridge between ZIF-8 and CelF to enhance the binding, a novel conductive ZIF-8/MO-PPy@CelF composite was successfully fabricated by simple in situ growth strategy. The increase in the deposition ratio of ZIF-8 on CelF from 7.8% (ZIF-8/CelF) to 31.8% (ZIF-8/MO-PPy@CelF) indicates that the MO-PPy generated in situ as a mediating layer is effective in promoting the growth of ZIF-8 on CelF. The factors influencing ZIF-8 deposition are mainly chemical dosages and time; temperature has a negligible effect. The morphology and structure of ZIF-8/MO-PPy@CelF composite were characterized by SEM, XRD, FTIR and XPS. The ZIF-8/MO-PPy@CelF was a positive triboelectric material as confirmed by the KPFM analysis. The surface polarity of CelF significantly increased after ZIF-8 nanoparticles were deposited on CelF, and positive triboelectricity increased with the increase in the deposition ratio of ZIF-8. Using ZIF-8/MO-PPy@CelF and PEFT as friction materials, a C-TENG based on contact-separation mode was fabricated. The C-TENG based on the ZIF-8/MO-PPy@CelF with 31.8% ZIF-8 deposition ratio produced a voltage output of 129 V, a current of 6.8 μA and a transfer charge of 47.4 nC, approximately four times higher than the C-TENG based on ZIF-8/CelF. Moreover, the C-TENG demonstrates excellent cycling stability after continuous operation for 10000 cycles. Our methodology provides insight to further expand the range of positive triboelectric materials available. C-TENG based on ZIF-8/MO-PPy@CelF composite is expected to be used in many applications such as self-powered supercapacitors, sensors and monitors, smart pianos, ping-pong tables, floor mats, etc.

## Data Availability

The authors confirm that the data supporting the findings of this study is available within the article.

## Supplementary Materials

The following supporting information can be downloaded at: https://www.mdpi.com/article/10.3390/polym14020332/s1, Figure S1: SEM images of ZIF-8/MO-PPy@CelF at different locations; Figure S2: Current densities of samples; Figure S3: Open circuit voltages at different humidity levels; Table S1: Output performance comparison of C-TENG; Video S1: The LED lights were successfully lit up by C-TENG.
