# Supplementary material for: Methyl Orange-Doped Polypyrrole Promoting Growth of ZIF-8 on Cellulose Fiber with Tunable Tribopolarity for Triboelectric Nanogenerator
Source: Polymers (Basel). 2022 Jan 14;14(2):332. doi: 10.3390/polym14020332 (PMC8781174; doi:10.3390/polym14020332)
Supplement: Supplementary file 1 [file polymers-14-00332-s001.zip › polymers-1522235-supplementary.pdf]

polymers

MDPI

*Supplementary Material*

# Methyl Orange-Doped Polypyrrole Promoting Growth of ZIF-8 on Cellulose Fiber with Tunable Tribopolarity for Triboelectric Nanogenerator

**Qiang Li, Xianhui An and Xueren Qian ***

Key Laboratory of Bio-Based Material Science & Technology, Northeast Forestry University, Ministry of Education, Harbin 150040, China; 2020115563@nefu.edu.cn (Q.L.); anxianh509@163.com (X.A.)
* Correspondence: qianxueren@nefu.edu.cn; Tel.: +86-13304642918.

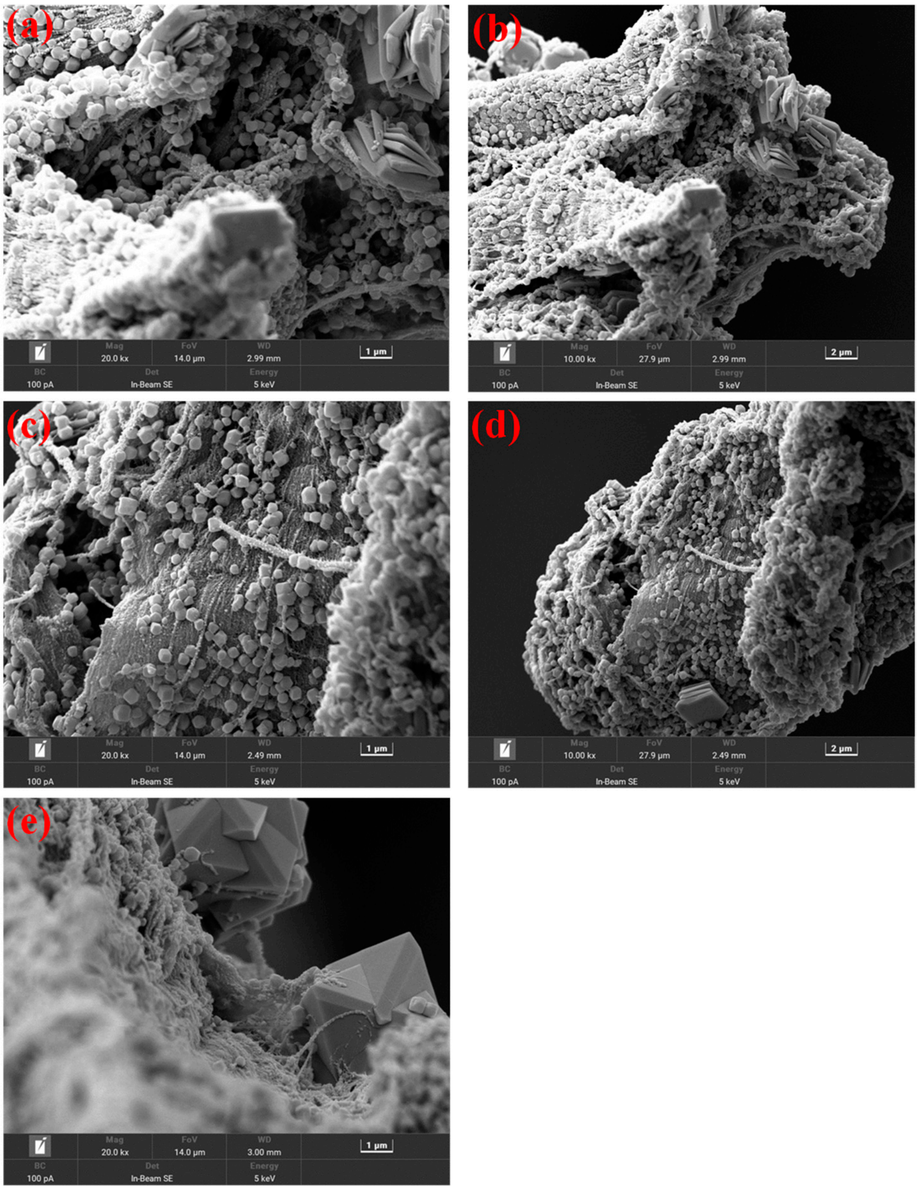


**Figure S1.** SEM images of ZIF-8/MO-PPy@CelF at different locations.

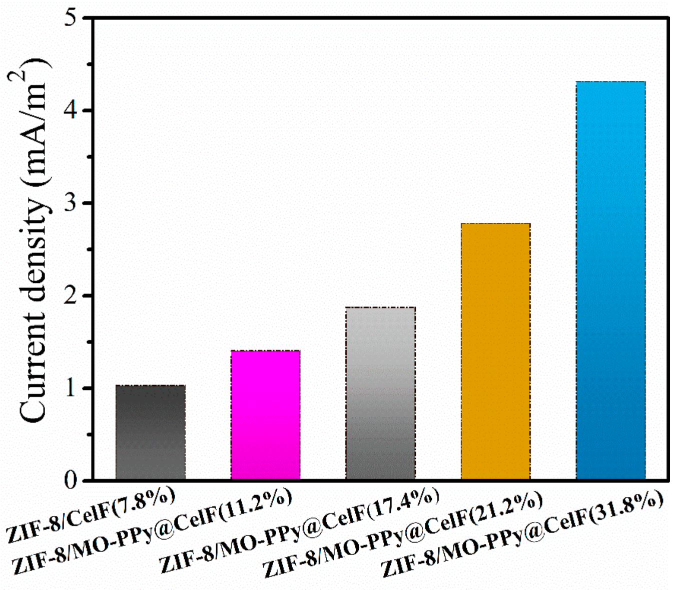


**Figure S2.** Current densities of samples.

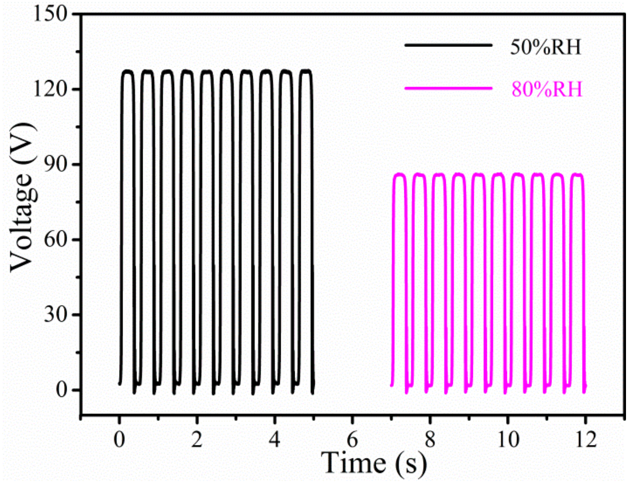


**Figure S3.** Open circuit voltages at different humidity levels.

**Table S1.** Output performance comparison of C-TENG.

| Positive Material | Negative Material | Voltage (V) | Current (μA) | Power Density (μW/cm²) | Reference |
|---|---|---|---|---|---|
| CNF-phosphorene | PET | 5.2 | 1.8 | 1.1 | 62 |
| Methyl-CNF | Nitro-CNF | 8 | 9 | - | 63 |
| CNF aerogel | PDMS aerogel | 60.6 | 7.7 | 450 | 64 |
| CNF-PEI aerogel | PVDF | 106.2 | 9.2 | 1330 | 65 |
| CNF-PEI-Ag | FEP | 100 | 1.1 | 43 | 66 |
| Alc-$S_5$-CNF | PVDF | 7.9 | 5.13 | 18.2 | 27 |
| Polyamide | PFOTES-CNF | 28.5 | 9.3 | 1.35 | 67 |
| ZIF-8/MO-PPy@CelF | PTFE | 129 | 6.8 | 3.33 (33.3 mW/m²) | **This work** |
